# Supplementary material for: The potential impact of human visceral leishmaniasis vaccines on population incidence
Source: PLoS Negl Trop Dis. 2020 Jul 2;14(7):e0008468. doi: 10.1371/journal.pntd.0008468 (PMC7363103; doi:10.1371/journal.pntd.0008468)
Supplement: Attachment — Submitted filename: epke review.docx [file pntd.0008468.s001.docx]

Reviewer Instructions

Thank you for agreeing to contribute your expert opinion to the evaluation of this manuscript.

**Assessing the manuscript**

As you evaluate the manuscript, consider whether it satisfies the [*PLOS NTDs* criteria for publication](https://journals.plos.org/plosntds/s/reviewer-guidelines#loc-criteria-for-publication):originality; importance and relevance to researchers, practitioners, or policy makers in the field of NTDs; interest for researchers or practitioners outside the field; rigorous methodology with conclusions justified by the evidence presented; and adherence to the highest ethical standards. Consult these resources for additional information:

- [Reviewer Guidelines](https://journals.plos.org/plosntds/s/reviewer-guidelines). An overview of our peer review process and what to consider in your review.
- [PLOS Reviewer Center](http://reviewers.plos.org/). Peer review how-to information and strategies.
- Other questions? Contact us at [plosntds@plos.org](mailto:plosntds@plos.org)

**Submitting your review**

To complete your review, answer the questions in the review form below. (A red asterisk (*) indicates that a response is required.) Declare any [competing interests](https://journals.plos.org/plosntds/s/reviewer-guidelines#loc-competing-interests) in the corresponding form question. This information is visible only to the editors and the journal office. Notify the journal if you have to recuse yourself from reviewing the manuscript.

**Confidentiality**

The review process is strictly confidential. If you become aware of any issue that could potentially compromise the integrity of the process, contact the journal before consulting with colleagues.

**Published peer review history**

If this manuscript is accepted for publication, the authors will have the option to publish the [peer review history](https://journals.plos.org/plosntds/s/editorial-and-peer-review-process#loc-peer-review-history) alongside the final article. The peer review history package includes the complete editorial decision letter, with the reviews, for each version of the manuscript, as well as the authors’ responses to reviewers.

**Anonymity**

Your identity will not be revealed to the authors unless you sign your review, or upload an attachment that has your name in the file name or contents. If you choose to sign your review, your name will appear in the editorial decision letter where the authors and other reviewers can see it. If the authors opt to publish the peer review history of their manuscript, the editorial decision letter will be published, including your signed peer review.

Review Questions

[Insert Special Character](https://www.editorialmanager.com/pntd/)

**Key Review Criteria Required for Acceptance?**

As you describe the new analyses required for acceptance, please consider the following:

**Methods**

-Are the objectives of the study clearly articulated with a clear testable hypothesis stated?
-Is the study design appropriate to address the stated objectives?
-Is the population clearly described and appropriate for the hypothesis being tested?
-Is the sample size sufficient to ensure adequate power to address the hypothesis being tested?
-Were correct statistical analysis used to support conclusions?
-Are there concerns about ethical or regulatory requirements being met?

(Limit 20000 Characters) [[Instructions]](javascript:__doPostBack('ctlReviewQuestions$Notes15$lbtnInstructions',''))

 
Character Count: 0

**Results**

-Does the analysis presented match the analysis plan?
-Are the results clearly and completely presented?
-Are the figures (Tables, Images) of sufficient quality for clarity?

(Limit 20000 Characters)

 
Character Count: 0

**Conclusions**

-Are the conclusions supported by the data presented?
-Are the limitations of analysis clearly described?
-Do the authors discuss how these data can be helpful to advance our understanding of the topic under study?
-Is public health relevance addressed?

(Limit 20000 Characters)

 
Character Count: 0

**Editorial and Data Presentation Modifications?**

Use this section for editorial suggestions as well as relatively minor modifications of existing data that would enhance clarity. If the only modifications needed are minor and/or editorial, you may wish to recommend “Minor Revision” or “Accept”. (Limit 20000 Characters)

 
Character Count: 0

**Summary and General Comments**

Use this section to provide overall comments, discuss strengths/weaknesses of the study, novelty, significance, general execution and scholarship. You may also include additional comments for the author, including concerns about dual publication, research ethics, or publication ethics. If requesting major revision, please articulate the new experiments that are needed. (Limit 20000 Characters)

 
Character Count: 0

**Confidential Comments to Editor**

Comment here if you have any editorial concerns that should be relayed confidentially to the editor and/or journal staff. Comments entered in this field will NOT be communicated to the authors. (Limit 20000 Characters)

 
Character Count: 0

***Competing Interests**

Do you have any potential or perceived competing interests that may influence your review? Please review our [Competing Interests policy](http://journals.plos.org/plosntds/s/competing-interests#loc-editors-and-reviewers)and declare any potential interests that you feel the Editor should be aware of when considering your review.

     

***Ethical Research and Reporting**

Has all research been conducted to a high ethical standard, and is the work performed the same work which was approved? (Note that PLOS NTDs staff runs an internal ethics check before papers proceed to review, so this is a secondary check.)

All research involving human participants must have been approved by the authors' Institutional Review Board or an equivalent committee, and all clinical investigation must have been conducted according to the principles expressed in the Declaration of Helsinki. Informed consent, written or oral, should also have been obtained from the participants. A brief overview of the ethics reporting should be provided in the Methods; if no consent was given, the reason must be explained.

     

If you responded “No” to the previous question, please briefly outline your concerns here: (Limit 20000 Characters)

 
Character Count: 0

***Data Availability**

Have the authors made all data underlying the findings in their manuscript fully available?

The PLOS Data policy requires authors to make all data underlying the findings described in their manuscript fully available without restriction, except in cases where the data are legally or ethically restricted (for example, participant privacy is an appropriate restriction).

     

***English Language**

Is the manuscript presented in an intelligible fashion and written in standard English?

     

If you responded “No” to the previous question, would additional copyediting make this manuscript acceptable for publication?

     

**Press Release**

If accepted, do you think this submission should be press released or receive additional media promotion on the basis of novelty, impact, and/or public interest?

     

*PLOS authors have the option to publish the peer review history of their article ([what does this mean?](https://journals.plos.org/plosntds/s/editorial-and-peer-review-process#loc-peer-review-history)). If published, this will include your full peer review and any attached files.

If you choose “no”, your identity will remain anonymous but your review may still be made public.

**Do you want your identity to be public for this peer review?** For information about this choice, including consent withdrawal, please see our [Privacy Policy](https://www.plos.org/privacy-policy).

     

ORCID Reviewer Credit

[What is ORCID?](javascript:%20showWhatIsOrcidDialog('pntd'))

[[Instructions]](javascript:__doPostBack('orcidInstructions',''))

* Do we have your consent to update your ORCID Record's Review Activity (subject to publication policy) to show that you completed a Review for this publication? This might trigger a one-time Authorization interaction with ORCID on proceeding.

Yes No
